# Lexical-Semantic Search Under Different Covert Verbal Fluency Tasks: An fMRI Study

**DOI:** 10.3389/fnbeh.2017.00131

**Published:** 2017-08-08

**Authors:** Yunqing Li, Ping Li, Qing X. Yang, Paul J. Eslinger, Chris T. Sica, Prasanna Karunanayaka

**Affiliations:** ^1^Department of Radiology, Pennsylvania State University College of Medicine Hershey, PA, United States; ^2^Department of Psychology and Center for Brain, Behavior, and Cognition, Pennsylvania State University University Park, PA, United States; ^3^Department of Neurosurgery, Pennsylvania State University College of Medicine Hershey, PA, United States; ^4^Department of Neurology, Pennsylvania State University College of Medicine Hershey, PA, United States; ^5^Department of Neural and Behavioral Sciences, Pennsylvania State University College of Medicine Hershey, PA, United States

**Keywords:** fMRI, neuropsychology, language, verbal fluency, semantics, phonemic, sentence completion

## Abstract

**Background:** Verbal fluency is a measure of cognitive flexibility and word search strategies that is widely used to characterize impaired cognitive function. Despite the wealth of research on identifying and characterizing distinct aspects of verbal fluency, the anatomic and functional substrates of retrieval-related search and post-retrieval control processes still have not been fully elucidated.

**Methods:** Twenty-one native English-speaking, healthy, right-handed, adult volunteers (mean age = 31 years; range = 21–45 years; 9 F) took part in a block-design functional Magnetic Resonance Imaging (fMRI) study of free recall, covert word generation tasks when guided by phonemic (P), semantic-category (C), and context-based fill-in–the-blank sentence completion (S) cues. General linear model (GLM), Independent Component Analysis (ICA), and psychophysiological interaction (PPI) were used to further characterize the neural substrate of verbal fluency as a function of retrieval cue type.

**Results:** Common localized activations across P, C, and S tasks occurred in the bilateral superior and left inferior frontal gyrus, left anterior cingulate cortex, bilateral supplementary motor area (SMA), and left insula. Differential task activations were centered in the occipital, temporal and parietal regions as well as the thalamus and cerebellum. The context-based fluency task, i.e., the S task, elicited higher differential brain activity in a lateralized frontal-temporal network typically engaged in complex language processing. P and C tasks elicited activation in limited pathways mainly within the left frontal regions. ICA and PPI results of the S task suggested that brain regions distributed across both hemispheres, extending beyond classical language areas, are recruited for lexical-semantic access and retrieval during sentence completion.

**Conclusion:** Study results support the hypothesis of overlapping, as well as distinct, neural networks for covert word generation when guided by different linguistic cues. The increased demand on word retrieval is met by the concurrent recruitment of classical as well as non-classical language-related brain regions forming a large cognitive neural network. The retrieval-related search and post-retrieval control processes that subserve verbal fluency, therefore, reverberates across distinct functional networks as determined by respective task demands.

## Introduction

Verbal fluency is often assessed using tasks that require participants to generate lists of words from long-term memory aloud under specific conditions and time constraints. They provide short and efficient screening instruments to evaluate access to and retrieval of verbal semantic knowledge (Birn et al., 2010; Lezak et al., 2012). Generating words according to a given rule relies on retrieval-related search and post-retrieval control processes such as organizing words into meaningful “clusters” and the flexibility to search and retrieve new clusters (Shao et al., 2014). Verbal fluency tasks have often been included in neuropsychological, clinical and non-clinical assessments investigating initiation, planning, lexical-semantic knowledge, lexical retrieval, articulatory flexibility, and executive control, etc. during language processing (Price, 2000; Costafreda et al., 2006; Hickok, 2009; Li et al., 2013). Therefore, verbal fluency tasks are used as efficient screening instruments for both verbal and executive control abilities (Henry and Crawford, 2004; Federmeier et al., 2010). Despite their widespread use, the anatomic and functional substrates of retrieval-related search and post-retrieval control processes of verbal fluency have not been fully elucidated.

Many studies on verbal fluency have employed either the phonemic fluency (sometimes referred to as letter fluency) or semantic fluency (sometimes referred to as category fluency) tasks during which participants deliberately generate words following either a phonological or a semantic cue (Strauss et al., 2006). While these tasks share several core processes such as accessing the mental lexicon, keeping in mind certain rules, maintaining focus on the task, and selecting words without repeating, they differ with respect to the kind of search processes required for successful retrieval (Martin et al., 1994; Fisk and Sharp, 2004; Unsworth et al., 2011). For example, phonemic fluency likely involves a serial search based on systematic syllabification of presented letters (Mummery et al., 1996; Rende et al., 2002). In contrast, category fluency is driven by association chains through which the search process is extended to encompass cue-related subcategories (Gruenewald and Lockhead, 1980). Additionally, category fluency also involves actively shifting between generated categories or subcategories (Rosen and Engle, 1997; Troyer et al., 1997; Reverberi et al., 2006). Semantic-category retrieval may very well require additional control processes such as selection of appropriate items from competing targets (Thompson-Schill et al., 1997, 1998). Therefore, it has been argued that the performance on phonemic and semantic fluency tasks may be differentially influenced by the lexical access ability (LAA) and executive control ability (ECA). The LAA is essential for the retrieval of grammatical representations and sound forms of words from the mental lexicon and the ECA is needed to control and regulate thought to direct behavior toward a general goal (Levelt et al., 1999; Shao et al., 2014). Research suggests that verbal ability is important for category fluency, while executive ability may play a greater role in letter fluency (Gruenewald and Lockhead, 1980; Rende et al., 2002; Shao et al., 2014).

Lexical-semantic access and retrieval have also been investigated using context-based fluency via sentence completion tasks (Duffy et al., 1989). Unlike single-word or word-pair stimuli in lexical association, lexical decision, verb generation, or picture naming tasks, sentence completion during context-based fluency requires the integration of peripheral information and context within a grammatical and communicative situation (Cohen and Servan-Schreiber, 1992; Cohen et al., 1999; Bazin et al., 2000; Brown et al., 2005; Fisher et al., 2007). Context maintenance involves delayed information processing that relies on the gist of the situation or task that usually requires greater cognitive control to guide later behavior. Although phonemic fluency, category fluency, and sentence completion tasks all share the need for lexical-semantic access and retrieval, they are thought to differ in their cognitive demands and the types of linguistic processing that occur. For example, sentence completion might be capitalizing on the context-based language system whereas phonemic and category fluency may be relying more on executive, verbal, and attentional abilities (Friederici, 2011; Shao et al., 2014). To investigate this proposal, the present study employed three covert word generation fMRI tasks in a sample of healthy, adult subjects to investigate similarities and differences among the Blood Oxygenation-Level Dependent (BOLD) activity patterns when guided by phonemic, semantic-category and fill-in–the-blank sentence completion cues. Further characterization and development of fMRI verbal fluency tasks will help refine our understanding of neuropsychological tests, and facilitate the development of imaging protocols that may eventually lead to reliable clinical applications (Birn et al., 2010; Morrison et al., 2016).

Context-based fluency may consist of several components (Rodriguez-Fornells et al., 2009; Friederici, 2012). For example, readers or listeners during sentence comprehension (or completion) must first retrieve the meaning of each individual word and combine them according to the sentence's syntactic structure to determine how the words relate to each other (Friederici, 2011). A plethora of neuroimaging studies have suggested a distributed network for lexical semantic processing including inferior parietal regions, temporal regions, and some regions related to category-specific (potentially sensorimotor-based) lexical representations (Hwang et al., 2009; Katzev et al., 2013). Although the left hemisphere is believed to mediate major language functions, right hemisphere contributions, likely related to context information, are well-documented (Beeman and Chiarello, 1998; Jung-Beeman, 2005; van Ettinger-Veenstra et al., 2010; Vigneau et al., 2011; Yang et al., 2016). Sentence comprehension is also subserved by morpho-syntactic processes involving inferior frontal regions (Marslen-Wilson and Tyler, 2007; Marslen-Wilson et al., 2008). Therefore, one goal of this study was to delineate prefrontal cortex (PFC) contributions, with an emphasis on the inferior frontal gyrus (IFG), encompassing both left-right hemispheres during verbal and context-based fluency task performance from a brain network perspective. Such information will help elucidate the brain basis of cognitive flexibility that subserves different word search strategies. A number of functional neuroimaging studies support the hypothesis that cognitive functions rely on distributed neural networks (Desmond and Fiez, 1998; Stowe et al., 1998; Papathanassiou et al., 2000; Bozic et al., 2010; Fonteneau et al., 2015; Rodd et al., 2015). Manipulation of selection demands by phonemic, category, and fill-in-the-bank sentence completion tasks, therefore, may lead to BOLD signal changes in brain regions that are not part of the classical language network (Vigneau et al., 2006, 2011).

Neuroimaging studies have implicated overlapping brain circuits encompassing the inferior frontal gyrus (IFG) in semantic-category and letter fluency tasks (Perani et al., 2003; Li et al., 2004; Costafreda et al., 2006; Hickok, 2009; Li, 2009; Yang and Li, 2012). The anterior-ventral region in the left IFG, i.e., the pars orbitalis of the left IFG (Brodmann Area [BA] 47), was found to be activated during category fluency task performance (Binder et al., 1997). In contrast, a posterior-dorsal region in the left IFG was activated during letter fluency task performance (Fiez, 1997). Similarly, the PFC has consistently been implicated as important for maintaining context as demonstrated by the use of the fill-in-the-blank sentence completion task in this study (Hagoort, 2005). Because PFC activity may be modulated by the hippocampus through the nucleus accumbens, frontal-lobe function during context-based information processing may also be associated with temporal-lobe function (Cohen and Eichenbaum, 1993; Grace, 2000). Although these regions are functionally interdependent, work on context-based information processing has suggested a clear distinction between timely semantic access and retrieval subserved by the frontal lobe and semantic memory representations stored in the posterior regions of the temporal cortex (Indefrey and Cutler, 2004; Tune and Asaridou, 2016; Tune et al., 2016).

Challenges, however, exist when conducting fMRI studies of verbal and context-based fluency. Covert designs have been used as an alternative to circumvent the task-related motion of overt speech that can cause signal artifacts in the frontal lobe (Birn et al., 1998, 1999; Gracco et al., 2005). Although covert designs have drawbacks, word generation studies have reported that the same brain regions were activated with greater intensity by the covert design when compared to the overt speech design (Birn et al., 2004; Hirshorn and Thompson-Schill, 2006). Therefore, we expected the single-letter, single-category, and fill-in–the blank sentence completion tasks to provide measures of efficiency for word retrieval based upon phonological, semantic, or contextually-cued information (Price, 2000).

In this study, we hypothesized that brain regions associated with covert word generation tasks would all be strongly left-lateralized in terms of the BOLD activation patterns (Thompson-Schill et al., 1997; Costafreda et al., 2006; Karunanayaka et al., 2010). Secondly, we expected that one or more “hot spots” of overlapping activation for all 3 tasks would be identifiable but that each task would also differ from the other two in aspects of their neural activation (Friederici, 2011). The sentence-completion task, relative to letter-fluency and semantic-category tasks, will lead to greater bilateral temporal BOLD activation (Menenti et al., 2009; Lam et al., 2016). The sentence completion task was also expected to elicit activation in a frontal-temporal network that included the right hemisphere (Vigneau et al., 2006; Dick et al., 2009, 2010). The phonemic fluency task was hypothesized to elicit greater left inferior frontal region activation when compared to the semantic-category task because of increased selection demands associated with retrieving words according to novel phonemic/spelling rules. In contrast, the category fluency task was expected to elicit greater occipital activation, reflecting the semantically associated word retrieval demands of category-specific tasks (Uchida et al., 1999). We used General Linear Model (GLM), Independent Component Analysis (ICA), and psychophysiological interaction (PPI) to identify and characterize the neural substrate of verbal fluency as a function of retrieval cue type based on phonological, semantic, or contextual information (Calhoun et al., 2001; Cisler et al., 2014). The identification of brain regions that facilitate the access of stored conceptual knowledge as a function of retrieval cue type will elucidate the functional substrates of retrieval-related search and post-retrieval control processes. Additionally, it will provide normative data to develop clinically relevant verbal fluency tasks for the accurate evaluation and localization of language function in individuals with focal as well as non-focal neurocognitive disorders (Monsch et al., 1992; Geurts et al., 2004; Henry and Crawford, 2004).

## Materials and methods

### Participants

Twenty-one native English-speaking, healthy, adult volunteers participated in the study (mean age = 31 years; age range = 21–45 years; 9 females). Educational level of participants ranged from a college bachelor's degree to a doctoral degree. Written informed consent was obtained from all participants and the research protocol had Penn State College of Medicine Internal Review Board approval. All participants were right-handed according to the Snyder and Harris' handedness questionnaire (Snyder and Harris, 1993) and had no history of neurological or psychiatric disorders. Additionally, all participants had normal reading/vocabulary levels (based on neuropsychological testing) with normal or corrected-to-normal vision acuity.

### Neuropsychological testing

Language and cognitive abilities of study participants were evaluated in terms of confrontational word retrieval ability using the Boston Naming standardized test before fMRI scanning (Kaplan et al., 1983). Confrontation naming is composed of several different processes: first, a subject must correctly perceive the object in the picture, second, he or she determines the associated semantic concept of the picture, and retrieves and expresses the specific, appropriate name for the object (del Toro et al., 2011). Specifically, the spontaneous response accuracy (i.e., the ability to correctly name objects spontaneously) was calculated for the sample. Stimulus cue response accuracy (ability to correctly name an object after a semantic cue) and phonemic cue response accuracy (ability to correctly name an object after a phonemic cue) were also tested for missed items.

### fMRI paradigms

**Figure 1:**
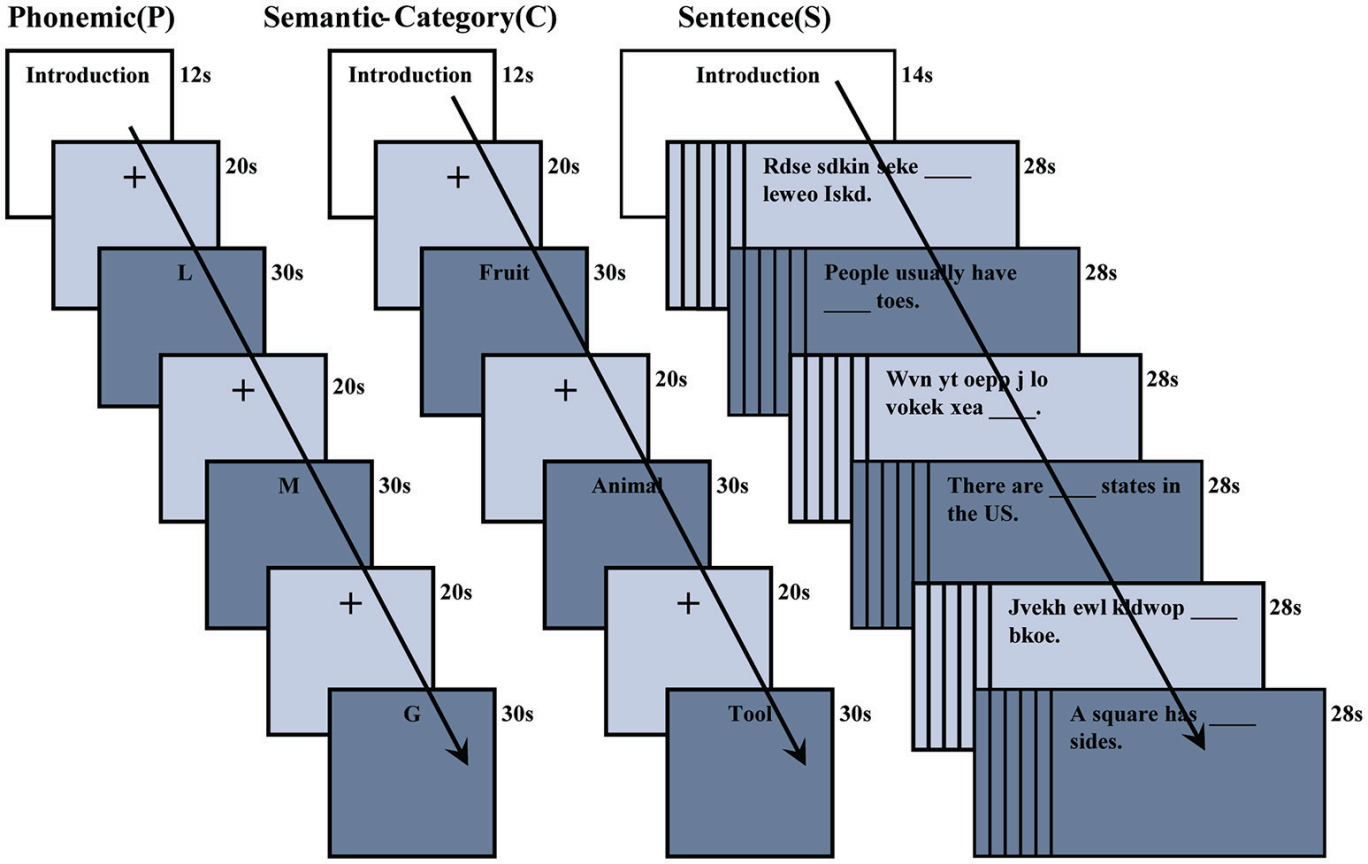
Phonemic (P), semantic-category (C), and sentence completion (S) verbal fluency fMRI tasks. A letter or semantic-category word was presented for 30 s during P or C tasks. The baseline condition for P and C tasks consisted of a cross fixation which was presented for 20 s. Sentences and pseudo-sentences were alternately presented for 28 s during the S task.

Phonemic fluency (P), semantic-category fluency (C), and fill-in-the-blank sentence completion (S) covert tasks, each placing different demands on the linguistic system, were implemented using a block design approach and presented in separate sessions. During fMRI scanning, participants had to covertly generate appropriate words depending on the task type (see Figure 1). Before scanning, all subjects were trained using shorter versions of the P, C and S paradigms. To ensure task performance, subjects were instructed to press a button after covertly generating a response during each fMRI task.

### Phonemic fluency (P)

During this task, participants were presented with three separate letters in sequential order. Each letter was displayed for 30 s followed by a fixation cross (baseline condition) for 20 s. The task required participants to silently generate words that begin with the displayed letter (for example, for the letter S, participants should covertly generate words such as “Sauce,” “Shoe,” “Sundae,” etc. until the letter disappeared from the screen); during the baseline condition, a fixation cross appeared on the screen and subjects were not required to perform any task.

### Semantic-category fluency (C)

During this task, participants were presented with three separate categories in sequential order. Each category word was displayed for 30 s followed by a fixation cross for 20 s as the baseline condition. The task required participants to silently generate words that belong to the same semantic-category once a category word was displayed (for example, for category “Fruit,” participants had to covertly generate words such as “Apple,” “Banana,” “Watermelon,” etc.); during the baseline fixation cross condition, subjects were not required to perform any task.

### Sentence completion (S)

During this context-based fluency task, participants were presented with either sentences or pseudo-sentences. The control condition included pseudo-sentences, in order to delineate the neural basis of lexical-semantic access via sentence completion tasks. Each sentence or pseudo-sentence condition was contained in a group of six sentences and was interleaved in separate sessions. Sentences contained missing words and were displayed for 28 s. Each sentence was followed by a fixation cross that was displayed for 500 ms. The task required participants to covertly generate a word to fill in the blank in every sentence (for example, “A doctor usually wears ___ clothes.” Participants had to generate a word such as “white” to complete the sentence). Pseudo-sentences (e.g., “Rdse sdkin seke_____ leweo iskd.”) contained missing words and displayed for 28 s. Each pseudo-sentence was followed by a fixation cross that was displayed for 500 ms, during which subjects were not required to perform any task (Moore-Parks et al., 2010).

### fMRI/MRI scanning parameters

Functional and structural MR images were acquired from all subjects at 3T (Siemens, Trio MR Scanner) with an 8 channel, phased-array head coil at the Pennsylvania State College of Medicine Hershey Medical Center. T2^*^-weighted gradient-echo EPI sequence with the following imaging parameters was used for fMRI image acquisition: TR = 2,000 ms; TE = 30 ms; flip angle = 90°; FOV = 240 × 240 mm; Matrix size = 80 × 80; voxel size = 3.0 × 3.0 × 4.0 mm; 35 interleaved axial slices; slice thickness = 4 mm with no slice gap; Number of time points: for the letter-word task = 162 and semantic-category task = 162 and for the sentence-completion task = 175. A high-resolution, T1-weighted MPRAGE scan was also obtained from each subject for functional overlay with the following parameters: TR = 2,300 ms; TE = 2.98 ms; flip angle = 90°; FOV = 256 × 256 mm, Matrix = 256 × 256 pixels, slice thickness = 1 mm with no slice gap; number of slices = 160 and voxel size = 1 × 1 × 1 mm. All fMRI paradigms were presented in E-PRIME^1^ using the Eloquence fMRI Software package (Invivo, Inc.: www.invivocorp.com).

### Data processing and analysis

fMRI data were processed using SPM12 software (http://www.fil.ion.ucl.ac.uk/spm/software/spm12/) by Matlab 8.0 (MathWorks, Natick, MA). Standard SPM12 steps were followed for fMRI image realignment, coregistration, and normalization to the Montreal Neurological Institute brain template (Collins et al., 1998). An activation map for each condition was generated using the GLM approach. Individual activation maps for P, C, and S tasks were then subjected to second-level, random-effects analysis to generate group-level statistical maps. A conjunction analysis was performed to demonstrate the context-invariant nature of regional responses during respective verbal fluency fMRI tasks (Friston et al., 2005). Group differences between P, C, and S tasks were evaluated using one-way ANOVA as implemented in SPM12. A regions-of-interest (ROI) analysis was implemented to examine group differences in terms of percent signal change in the left hemisphere temporal regions during the S task.

An additional ROI analysis was performed to further examine differential fMRI activation patterns in the left and right temporal regions during the S task as follows. We selected the coordinates of the peak activated voxel (*p* < 0.001, cluster size = 125) for a given temporal brain region using Table 1. A sphere with a 6 mm radius was then defined as the ROI using the coordinates of the peak activated voxel as the center of the sphere. This ROI was then used to extract the mean percent signal change for each subject followed by averaging across participants to obtain the group mean percent signal change for a given temporal brain region during the S task. The group mean percent signal change values were compared to investigate differential activation patterns in the left and right temporal regions during the S task.

**Table 1:** Brain regions with significant activation for Phonemic (P), Semantic-category (C), and Fill-in-the-blank Sentence completion (S) verbal fluency fMRI tasks.

**Phonemic(P)**									**Semantic-Category(C)**									**Sentence(S)**								
**Regions activated**		**L/R**	**MNI coordinates**			**BA**	**T**	**K_€_**	**Regions activated**		**L/R**	**MNI coordinates**			**BA**	**T**	**K_€_**	**Regions activated**		**L/R**	**MNI coordinates**			**BA**	**T**	**K_€_**
			**X**	**Y**	**Z**		**score**	**voxels**				**X**	**Y**	**Z**		**score**	**voxels**				**X**	**Y**	**Z**		**score**	**voxels**
Frontal	Superior	L	−32	50	20	6	3.4	37	Frontal	Superior	L	−30	48	24	8	6	44	Frontal	Superior	L	−16	34	52	6	3.7	351
	Superior	R	26	48	−8	9	4.5	4		Superior	R	6	4	68	6	8.5	1		Superior	R	24	58	34	6	6.2	171
	Middle	R	30	42	26	9	5.2	515		Medial	R	42	40	38	9	4.7	265		Medial	L	−22	40	−16	6	4.1	194
																			Medial	R	48	42	20	11	3.6	334
	Inferior	L	−52	−2	52	44	9.9	1420		Inferior	L	−37	24	−4	47	4.9	88		Inferior	L	−44	16	18	44	12	489
	Inferior	R	42	16	2	45	7.5	131		Inferior	R	38	36	26	47	4.9	80		Inferior	R	32	40	−18	47	3.9	164
SMA		L	−46	2	40	4	8	490	SMA		L	−34	−18	72	4	3.1	278	SMA		L	−4	−34	68	4	112	144
SMA		R	58	2	46	4	5.3	107	SMA		R	42	8	30	4	3.4	144	SMA		R	52	4	52	4	5.1	166
ACC	Cingulate	L	−2	12	50	32	11	153	ACC	Cingulate	L	−4	10	52	32	4.7	17	ACC	Cingulate	L	0	6	24	32	3.8	83
Insula		L	−40	4	26	−	8.9	15	Insula		L	−24	20	20	−	3.1	27	Insula		L	−46	14	4	−	11	149
									Occipital	Lingual	L	−10	−98	−6	18	7	42	Occipital	Cuneus	L	−44	−64	20	17	3.6	72
										Lingual	R	16	−88	−10	18	4.1	16		Lingual	R	2	−90	−4	17	12	82
																		Temporal	Superior	L	−58	−44	12	22	12	382
																			Superior	R	40	20	−20	38	5.7	217
																			Middle	L	−50	−50	12	21	12	299
																			Middle	R	46	24	−10	21	7.6	10
																			Inferior	L	−48	−70	−1	19	2.3	
																			Inferior	R	38	−42	−24	20	3.6	28
																		Parietal	Postcentral	L	−28	−70	50	4	13	28
																			Precuneus	R	26	−52	42	7	6	72
																		Parahippocampal		L	−20	−28	−4	27	12	78
																		Thalamus		L	−14	−8	10	−	5	65
																		Cerebellum		L	−2	−28	−22	−	3.9	50
																				R	10	−76	−24	−	15	195

*L, left; R, right; BA, Brodmann's area; SMA, supplementary motor area; ACC, anterior cingulate cortex. The coordinates of the peak activated voxel T-value, for each contrast cluster, was selected as the center of the ROI defined as a sphere with a 6-mm radius*.

The psychophysiological interaction (PPI) was used to further investigate whether the connectivity between the left superior temporal regions and frontal regions was modulated during the S task (Friston et al., 1997; O'Reilly et al., 2012; Cisler et al., 2014). The ROI for the left superior temporal region was generated using SPM results of the S task and the frontal regions were generated using the AAL atlas (Tzourio-Mazoyer et al., 2002). In the PPI linear regression model, the task on-off reference time course, temporal region time course, and the PPI effects between them were added as independent variables along with a constant. A significant interaction effect would imply that the connectivity between the temporal and frontal regions was modulated by the two types of trial conditions in the S task.

In order to investigate brain networks, both lateralized and bilateral that subserve context-based verbal fluency during the S task, ICA was utilized as detailed in several previous publications (Calhoun et al., 2001; Karunanayaka et al., 2007, 2010). The rationale to use ICA was based on research suggesting that language comprehension is subserved by a distributed bilateral system as well as left hemisphere (LH) frontotemporal systems (Marslen-Wilson and Tyler, 2007). For the purpose of completeness, a brief description of the ICA methodology is provided in the following section. ICA estimation was preceded by several data preprocessing steps [e.g., (1) converting to percent signal changes; (2) principal component analysis (PCA) related data reduction (subject-wise and group-wise); (3) data concatenation across subjects, etc.]. ICA decomposition was based on the multiple runs of the FastICA algorithm followed by agglomerative clustering (Hyvarinen, 1999; Himberg et al., 2004). Individual IC maps were obtained using back propagation methods described in Calhoun et al. (2001). The group IC maps were calculated using a voxel wise one-sample *t*-test on individual IC maps obtained in the previous step. To determine the task-relatedness of each group map, individual IC time courses were Fourier-transformed (FT) and the component at the on/off task frequency was subjected to a *post-hoc* analysis (Karunanayaka et al., 2007, 2010).

## Results

### Behavioral results

The accuracy rates for the test of confrontational word retrieval were as follows: the spontaneous accuracy rate was 86% ± 9 and the combined cued response correct rate (stimulus cues + phonemic cues) was 77% ± 19. Although the spontaneous accuracy rate was higher than the stimulus cue accuracy rate, the differences were not statistically significant (*p* > 0.05). The average total score, i.e., spontaneous + stimulus cues + phonemic cues, was 94% ± 6.

### The whole brain activation

**Figure 2:**
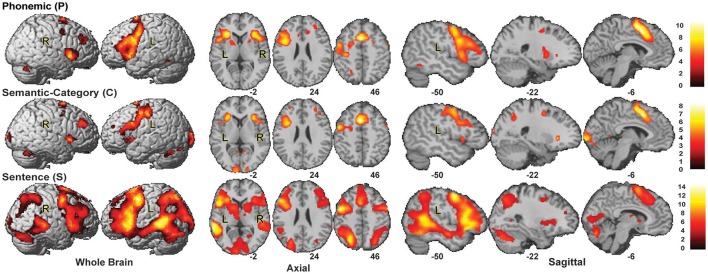
Group Activation maps for P, C, and S tasks (*p* < 0.001, cluster size = 125). The activation pattern suggested a graded distinction between tasks in the left IFG (LIFG).

The group activation maps for the P, C, and S tasks (*p* < 0.001, cluster size = 125) are shown in Figure 2. The number of activated brain regions progressively increased from P to C and to S. Activated brain regions and their activation foci are tabulated in Table 1.

**Figure 3:**
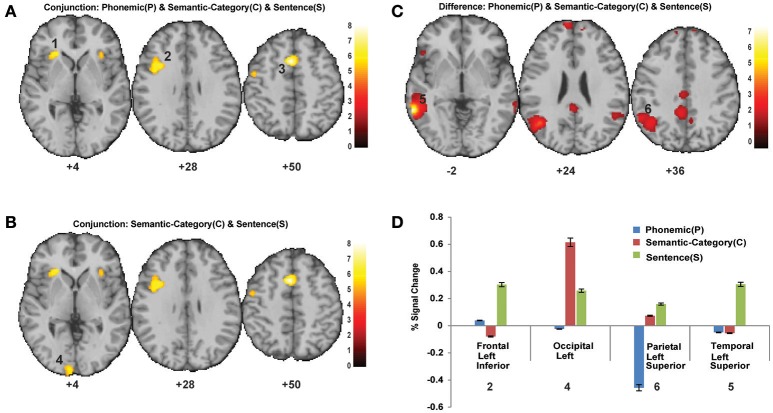
**(A)** Conjunction analysis for P, C, and S tasks (*p* < 0.05, FWE). Significant activation was detected in the bilateral frontal cortex, bilateral precentral gyrus. **(B)** Conjunction analysis for C and S tasks (*p* < 0.05, FWE). Significant activation was detected in the bilateral occipital cortex in addition to activated regions in **(A)**. **(C)** Activation difference map for P, C, and S tasks (*p* < 0.05, FWE). Left inferior frontal, left superior temporal, and left parietal showed activation differences between P, C, and S tasks. **(D)** Percent (%) signal change in brain regions that showed differential activation for P, C, and S tasks.

Conjunction analysis for the P, C, and S tasks revealed that there were common areas of activation across all 3 tasks (Figure 3A). Common activation clusters were detected in the left superior frontal lobe (peak MNI coordinates: −36, 12, 28), the bilateral inferior frontal lobe (peak MNI coordinates: −26, 26, −2), and the bilateral SMA (peak MNI coordinates: −4, 12, 50; *p* < 0.05 FWE). Similarly, conjunction analysis for C and S tasks revealed common activation in the left occipital (peak MNI coordinates: −8, −98, −4) regions (Figure 3B) in addition to activations detected for the conjunction of P, C, and S (*p* < 0.05 FWE). Conjunction analysis for P and S (data not shown) resulted in a very similar activation pattern to that of the conjunction analysis of P, C, and S.

In addition to overlapping neural substrate for these tasks, distinctive activations were also found. Figures 3C,D summarize the activation differences between P, C, and S tasks. Differential activation was detected in the left inferior frontal (peak MNI coordinates: −50, 26, 0), left superior parietal (peak MNI coordinates: −46, −62, 26), and left superior temporal (peak MNI coordinates: −62, −46, −2) regions at *p* < 0.05, FWE. The differences in left occipital (peak MNI coordinates: −10, −100, −5) regions were detected at *p* < 0.001, uncorrected. Except for the left occipital region, the BOLD percent signal change was higher in other regions during the S task (Figure 3D).

### ROI

**Figure 4:**
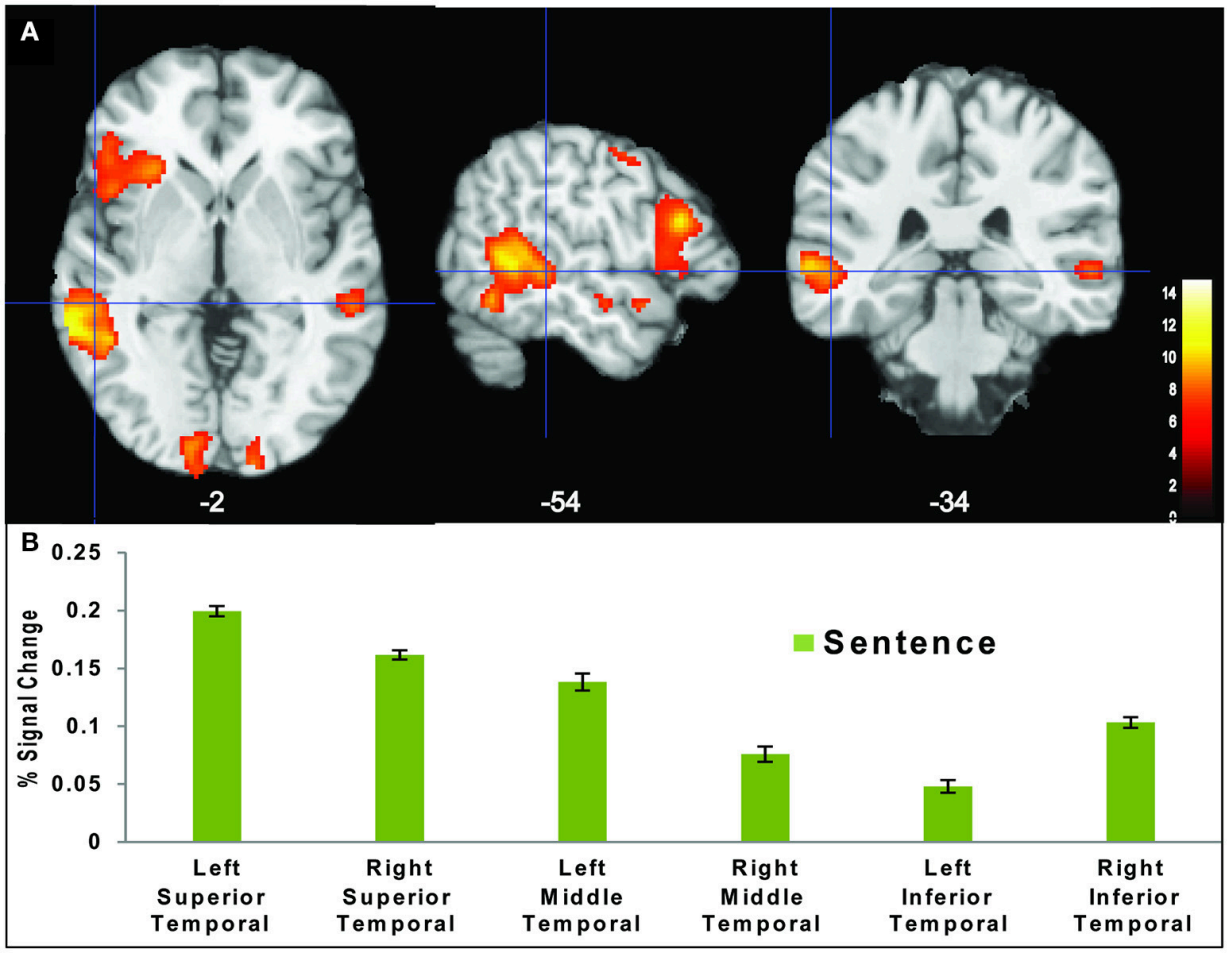
**(A)** Temporal region activation during the S task (*p* < 0.001). **(B)** Region of interest analysis (ROI) showing the average percent (%) signal change in temporal regions.

The ROI analysis in the posterior-superior temporal lobe (Figure 4), highlighting BOLD activation differences in terms of percent signal change, revealed that the grammatical nature of the S task was very powerful in activating both the left and right temporal regions in comparison to the P and C tasks. This finding was unique to the S task.

### PPI

**Figure 5:**
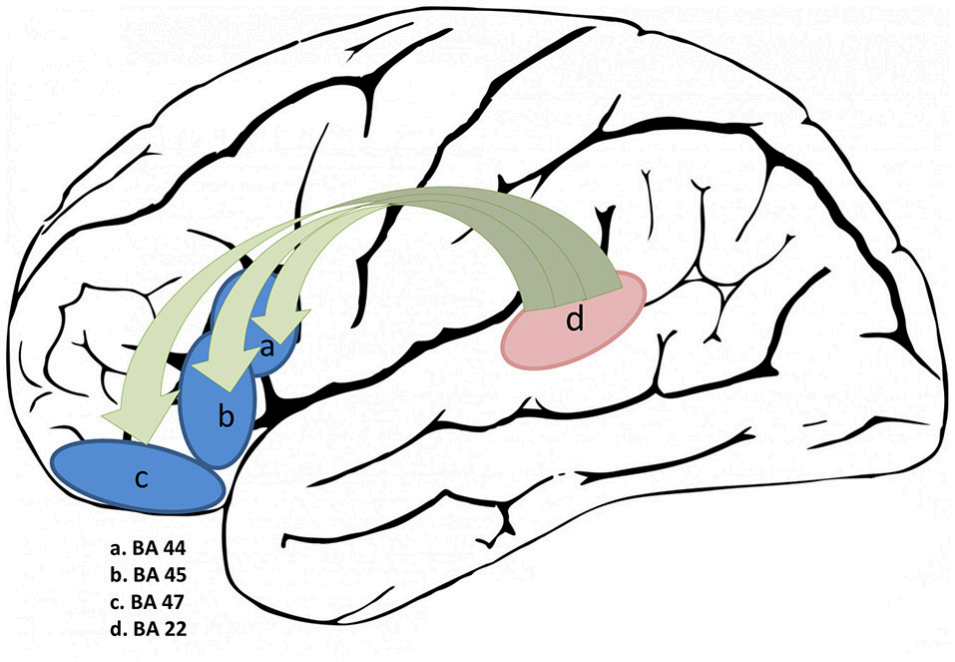
PPI connectivity analysis of frontal regions with the left superior temporal as the seed region.

The PPI analysis shown in Figure 5 revealed that the connectivity between the left superior-posterior temporal (BA 22) and frontal regions (BA44, BA45, and BA47) was significantly different (*p* < 0.0001) between sentence and pseudo-sentences trial conditions (Table 2). As such, it appears that the left superior temporal region tends to increase the contrast between the effects of the two trial conditions on frontal regions.

**Table 2:** PPI connectivity analysis for the S task.

						**Left superior temporal (−60, −48, 12)**
	**R/L**	**X**	**Y**	**Z**	**BA**	***P***
Frontal-Inferior	L	−52	6	10	44	0.003
Frontal-Inferior	L	−38	30	0	45	0.003
Frontal-Inferior	L	−22	32	−10	47	0.002
Hippocampus	L	−24	−34	−4		0.001
Parahippocampus	L	−22	−4	−24		0.003

*Statistically significant connectivity modulations were detected between left superior temporal and the left frontal regions. L, left; BA, Brodmann's area*.

### ICA

**Figure 6:**
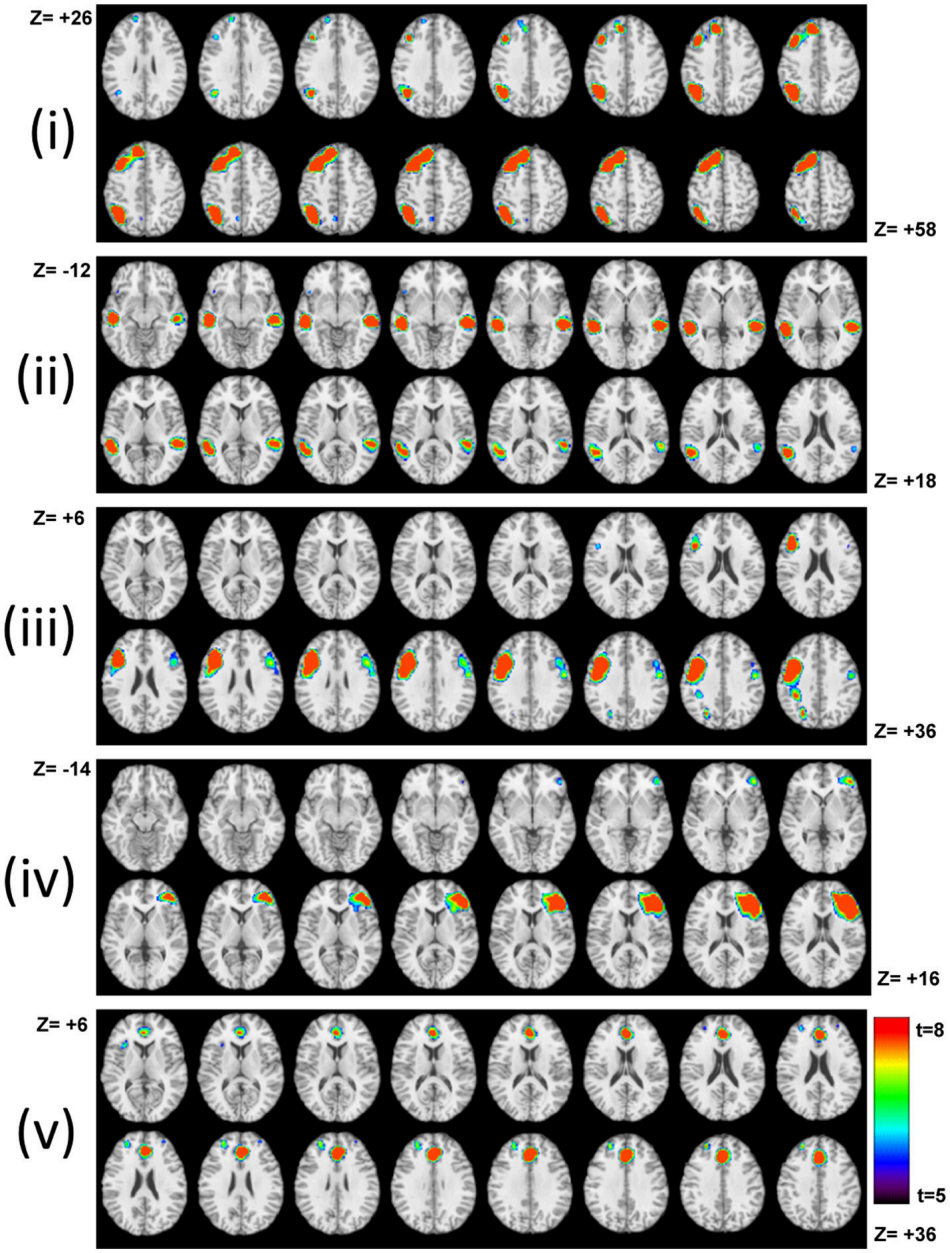
Task–related IC networks that subserve the S task. Activated Networks can be identified as left, bilateral, or left dominant. They encompass **(i)** left frontal-parietal regions; **(ii)** bilateral temporal regions; **(iii)** left frontal regions; **(iv)** right frontal regions, and **(v)** the anterior cingulate. All maps are in neurological convention. The results highlight the significance of right hemisphere involvement in the S task.

Five task-related IC maps consisting of the frontal, temporal, and parietal regions presumed to subserve lexical semantic processing, including search and retrieval during the S task, are shown in Figure 6. ICA analysis detected additional task-related brain networks showing correlated activity across distributed brain regions that the GLM method was unable to resolve. The involvement of the right frontal–parietal regions in sentence processing likely reflects engagement of the domain general cognitive control network depending on task demands at different points in a sentence (Lam et al., 2016). Table 3 tabulates the respective correlation coefficients of each network shown in Figure 6. Therefore, the ICA results support earlier claims that language (sentence) processing recruits areas distributed across both hemispheres that extend beyond the classical language areas (Lam et al., 2016).

**Table 3:** The correlation coefficients of IC maps shown in Figure 6 with the task on-off reference function.

**IC component**	**Correlation with the task reference function**
i	0.35
ii	0.49
iii	0.63
iv	0.45
v	0.52

*All were significant at p < 0.0001. IC, independent component*.

## Discussion

While the feasibility of the phonemic fluency (P), semantic-category fluency (C), and context-based sentence completion (S) paradigms has been demonstrated before (Birn et al., 2010; Moore-Parks et al., 2010), this study focused on comparing and contrasting brain network activation patterns of P, C, and S tasks in relation to task demands in the same group of subjects and within a single imaging session. Overall, the results helped delineate PFC and IFG contributions to verbal and context-based fluency, including left and right hemispheres. In line with our hypotheses: (1) P and C tasks predominantly recruited frontal regions in the left hemisphere as part of the well-known language network (Vigneau et al., 2006) and (2) the S task recruited extensive bilateral frontal cortex (including SMA), bilateral temporal lobe, parietal cortex, occipital lobe, left insula and anterior cingulate, thalamus, parahippocampal, and cerebellar regions (Fedorenko and Thompson-Schill, 2014; Hagoort and Indefrey, 2014; Friederici and Singer, 2015). The context-based S fluency task requires word recognition and comprehension, the understanding of syntactic–semantic relationships between words, planning of a sentence structure and word retrieval to name a few (Garrett, 1980; Moore-Parks et al., 2010). Therefore, the activation and connectivity patterns observed during the S task is consistent with studies that have suggested a distributed brain network for lexical semantic processing encompassing multiple peri-sylvian structures including the inferior frontal gyrus, superior and middle temporal regions, inferior parietal regions, and some regions related to category-specific (potentially sensorimotor-based) lexical representations (Martin et al., 1994; Hwang et al., 2009). Results of this study suggest that lexical-semantic access and retrieval via fill-in-the-blank sentence completion is mainly subserved by brain networks implicated in contextual integration (i.e., context-based language system). On the other hand, as discussed in Shao et al. (2014), phonemic and category fluency may be predominantly subserved by executive, verbal, and attentional abilities (i.e., LAA and ECA abilities; Shao et al., 2014).

The P and C tasks appear to require many of the same cognitive processes such as sustaining attention, devising a search strategy, selecting appropriate words, inhibiting competitors, engaging working memory, and articulating the output (Shao et al., 2014). There are, however, important differences between the two in terms of utilizing LAA and ECA abilities for search strategies (Thompson-Schill et al., 1997). For example, the P task requires selecting and retrieving information based on phonological and morphological characteristics and in contrast, the C task places greater demand on linguistic-conceptual knowledge (Vigneau et al., 2006). Our results partly agreed with this delineation between LAA and ECA abilities and therefore, cognitive mechanisms and brain resources for lexical search in the respective tasks. This is because the P task showed greater activation within the left frontal lobe, especially in the more posterior regions of the left IFG (Costafreda et al., 2006; Katzev et al., 2013). This study, however, did not support previous studies that showed increased activation in posterior regions of the temporal cortex for the C task. This may be due to inconsistent individual activation patterns that we observed in the temporal lobe, which is likely due to the low burden required of comprehension for these tasks (Gaillard et al., 2003). The C task, when compared to P task, showed greater activation in the occipital cortex. The occipital cortex may place an extra emphasis on visual cues to identify a whole word and then to retrieve the word from the corresponding brain network (Petersen et al., 1990). C task, therefore, might involve a mechanism to map the whole word to the verbal store directly unlike the P task where an initial mapping of the letter cue to phonologic and/or orthographic information may be required.

Greater activation was detected in the right medial/inferior frontal gyrus and left parietal cortex during the S task compared to P and C tasks. Moreover, the activation extended beyond Broca's area into several adjacent motor-related regions. Sentence processing during context-based fluency involves morpho-syntactic processes that go beyond the word level and, as a result, frontal regions, especially the inferior frontal regions, are considered critically important for sentence comprehension (Marslen-Wilson and Tyler, 2007). We also detected greater activation in left temporal regions during the S task that included superior, middle, and inferior temporal gyri that may be related to the richness and complexity of semantic representations during sentence comprehension (Blumenfeld et al., 2006). This observation also supports the assertion that brain activation in these regions will increase with semantic complexity, i.e., semantic search and selection based on contextual information to complete each sentence. The PPI analysis revealed a significant connectivity between the left superior temporal and frontal regions during sentence completion. The pattern of activation and connectivity of these tasks may explain certain clinical observations in patients with temporal and frontal lobe damage (Butters et al., 1986; Chan et al., 1993; Hodges et al., 1999; Baldo et al., 2006). For example, it has been shown that frontal lobe damage causes considerable difficulties during phonemic fluency tasks (Baldo et al., 2001), while temporal lobe damage affects S fluency to a greater extent (Baldo et al., 2006). Our results suggest that the contrast is not between P and C tasks, but between the S task on the one hand and P and C tasks on the other, because of stronger temporal cortex activation for lexical-semantic access and retrieval during the S task.

We also detected activation in the precentral gyrus (SMA, BA 4) that has been observed during previous language-related fMRI tasks (Gaillard et al., 2003) and is often assumed to be due to response-related motion, such as overt speech or button press. Since our study involved covert speech, we hypothesize that the observed activation in SMA may be due to “inner speech” (Wise et al., 1991; Chee et al., 1999). Verbal working memory is inherent in most language tasks as participants need to hold a word “online” while making a decision or generating a response. The S task required the participant to keep the definition of the sentence online while processing the middle words of the sentence. SMA involvement in sequence movement generated from memory has been well established (Shima and Tanji, 1998). The anterior cingulate (ACC) was also activated during P, C, and S tasks. ACC is usually activated in visual stimulus orientation search/selection and semantic attention tasks, and the activation is positively correlated with increased conflict monitoring of targets while inhibiting competing targets (Fan et al., 2008). Based on our study results, the ACC activation was greater during the P and C tasks when compared to the S task. Thus, we hypothesize that search and decision making about an appropriate word from competing candidates during P and S tasks demand more executive control resources than the C task. The S task also generated higher BOLD activation in the bilateral cerebellum which is consistent with previous studies that reported cerebellum participation in a wide range of non-motor tasks such as learning, working memory, and language (Desmond and Fiez, 1998). Additionally, we also found the thalamus, parahippocampal area, and insula to be associated with the S task, which highlights that syntactic and semantic information processing may be primarily realized within cortico-thalamic networks (Hebb and Ojemann, 2013).

The ICA analysis of the S task identified right dominant networks in addition to classic left-dominant language networks encompassing the IFG and middle temporal gyrus (Kircher et al., 2001). The contribution of the right hemisphere (homolog to classical language areas) for language processing has been associated with increased task demand such as decoding ambiguity, metaphors and distant semantic relationships (Just et al., 1996; van Ettinger-Veenstra et al., 2010). Our ICA results showed that sentence completion as part of context-based fluency is complex enough to involve morpho-syntactic processes and engage the right hemisphere involvement (Marslen-Wilson and Tyler, 2007). The left parietal and prefrontal involvement in sentence processing suggested that verbal material in the S task may require the phonological loop to a greater extent than the easy-to-perform P or C tasks (Jonides et al., 1998; Baddeley et al., 1999; Logie et al., 2003). At the same time, the lack of right parietal involvement in the S task implied that the left hemispheric language system was not overtaxed by the word retrieval demands during the S task (Drager et al., 2004). In general, difficult-to-perform word searches require sustained attention, working memory, executive response selection, and control resulting in an increase in right hemispheric activation associated with networks such as attention and executive control (Drager et al., 2004).

According to ICA formalism, an ICA map can be assigned functional modularity presumed to subserve a unique aspect of language processing in the human brain (Calhoun et al., 2004). Furthermore, group ICA methods can detect additional activation foci that are not detected by the standard GLM methods. This is due to activity being shifted temporally and delayed with respect to the hemodynamic response function (HRF) (Tie et al., 2008). Spatial ICA has the ability to separate source signals from a given brain region and provide new insight into its functional significance otherwise hidden from model based GLM techniques. ICA and PPI results of this study suggest that a more complex and parallel network was recruited for sentence completion. The implications of this new information, however, cannot be conclusively determined based on the current experimental design.

Taken together, our results highlighted the importance of right hemisphere contributions in sentence/text comprehension and decisions about semantic relations between words to sentence-level language processing (Kaplan et al., 1990; Faust and Chiarello, 1998; Vigneau et al., 2011; Yang et al., 2016). This study also identified differential patterns of brain activation for P, C, and S tasks which were clearly linked to respective task demands. Our results did not support clear dissociations in the left temporal regions or the left IFG on the basis of linguistic information that each task processes (Friederici, 2012). The importance of the left posterior temporal lobe and its connectivity to left IFG for sentence completion, i.e., context-based fluency, was emphasized by the results of the S task. Results also suggest a graded distinction between respective fluency tasks in the left IFG with a clear overlap in BA44. This may be interpreted as supporting substantial overlap in function as well as the interactions between retrieval-related search and post-retrieval control processes that subserve verbal and context-based fluency tasks used in this study (Hagoort, 2005). Nevertheless, this study provided new insight into how task demands modulate the neural substrate of verbal fluency. This insight will have significant theoretical and practical implications in understanding the organization of the language network in the human brain (Xu et al., 2013).

### Study limitations

A general limitation of silent word generation fMRI schemes is the lack of behavioral recordings (Fu et al., 2002). The limited number of neuropsychological tests performed in this study prevented us from conducting detailed brain-behavior correlation analyses. Future studies, therefore, should investigate relationships such as verbal and executive ability and participants' cue-dependent fMRI activity patterns. The relatively short number of blocks in the P and C tasks precluded a detailed ICA study to investigate the underlying cognitive modules of phonemic and category fluency. Cross-validation of the current findings using larger and independent datasets is, therefore, warranted to better characterize the anatomic and functional substrate of word retrieval during verbal fluency tasks (Lezak et al., 2012).

## Conclusion

The experimental design of this study provided a foundation to compare and contrast brain activation patterns during P, C, and S tasks and offered a well-grounded framework to test claims in the literature of left IFG and left temporal region functional segregation and integration on the basis of linguistic information (Friederici, 2012). This study demonstrated the utility of covert fMRI designs to delineate the neural substrate of verbal fluency by highlighting the recruitment of common as well as distinct brain regions when the search was guided by different retrieval cues (Li et al., 2004). The study revealed the importance of temporal regions for sentence comprehension and also supported the idea that lexical representations might be stored in the brain separately from medial temporal lobe structures (Rodriguez-Fornells et al., 2009). An alternative explanation might be that retrieval-related search and post-retrieval control processes during context-based fluency entails creating a type of gist semantic representation that can be independent of the specific verbatim lexical representations (Brewer, 1977; Graesser et al., 1994; Rodriguez-Fornells et al., 2009). The activation pattern during C and S tasks highlighted the importance of occipital regions in identifying whole words, while the S task activated an extended brain network that was hypothesized to subserve lexical semantic access during contextual information processing. The S task also revealed the contributions of the right hemisphere for language processing (Menenti et al., 2009; Bambini et al., 2011; Yang et al., 2016). Overall, the study results indicated that both the location and amount of cortical activity can be modulated by task demands depending on retrieval cue types. Rather than increasing activation in typical language-related areas, word search and retrieval during context-based fluency (i.e., processing beyond single word) leads to coactivation of distinct, bilateral brain areas that work together as part of a large cognitive network (Menenti et al., 2009). Finally, the study results may provide a basis to further develop fMRI paradigms to evaluate more distinctive processes of lexical retrieval that encompass executive function demands as well. Potentially, this will help distinguish clinical subjects from healthy subjects using activity in brain regions that are responsive to the various verbal fluency tasks presented in this study.

## Author contributions

YL, PE, and PK: Design, analysis, and writing. PL: Analysis and writing. QY: Design, data collection, analysis, and writing. CS: Data collection and writing.

### Conflict of interest statement

The authors declare that the research was conducted in the absence of any commercial or financial relationships that could be construed as a potential conflict of interest.

## Footnotes

^1^Psychology Software Tools, Inc. [E-Prime 2.0]. (2012). Available online at: http://www.pstnet.com.
